# Causal relationships exist between polycystic ovary syndrome and adverse pregnancy and perinatal outcomes: a Mendelian randomization study

**DOI:** 10.3389/fendo.2024.1327849

**Published:** 2024-06-28

**Authors:** Yuanlin Ma, Jiahao Cai, Lok-Wan Liu, Tianrui Wen, Weina Huang, Wenhui Hou, Zixin Wei, Yan Xu, Yanwen Xu, Yizi Wang, Qingyun Mai

**Affiliations:** ^1^ Reproductive Medicine Center, The First Affiliated Hospital, Sun Yat-sen University, Guangzhou, Guangdong, China; ^2^ The Key Laboratory for Reproductive Medicine of Guangdong Province, The First Affiliated Hospital of Sun Yat-sen University, Guangzhou, Guangdong, China; ^3^ Guangdong Provincial Clinical Research Center for obstetrical and gynecological diseases, The First Affiliated Hospital of Sun Yat-sen University, Guangzhou, Guangdong, China; ^4^ Department of Neurology, Guangzhou Women and Children’s Medical Center, Guangzhou Medical University, Guangzhou, Guangdong, China; ^5^ Reproductive Medicine Center, The First Affiliated Hospital of Zhengzhou University, Zhengzhou, Henan, China; ^6^ Department of Pulmonary and Critical Care Medicine, Sun Yat-sen Memorial Hospital, Sun Yat-sen University, Guangzhou, Guangdong, China

**Keywords:** polycystic ovary syndrome, adverse pregnancy and perinatal outcomes, genetic role, Mendelian randomization, hypertensive disorders of pregnancy, gestational hypertension

## Abstract

**Introduction:**

Previous observational studies have shown that polycystic ovary syndrome (PCOS) was associated with adverse pregnancy and perinatal outcomes. However, it remains controversial whether PCOS is an essential risk factor for these adverse pregnancy and perinatal outcomes. We aimed to use instrumental variables in a two-sample Mendelian randomization (MR) study to determine causality between PCOS and adverse pregnancy and perinatal outcomes.

**Materials and methods:**

Summary statistics were extracted from a recent genome-wide association study (GWAS) meta-analysis conducted in PCOS, which included 10,074 cases and 103,164 controls of European ancestry. Data on Adverse pregnancy and perinatal outcomes were summarized from the FinnGen database of European ancestry, which included more than 180,000 samples. The inverse variance weighted (IVW) method of MR was applied for the main outcome. To assess heterogeneity and pleiotropy, we conducted sensitivity analyses, including leave-one-out analysis, weighted median, MR-PRESSO (Mendelian Randomization Pleiotropy RESidual Sum and Outlier), and MR-Egger regression.

**Results:**

Two-sample MR analysis with the IVW method suggested that PCOS exerted causal effects on the risk of hypertensive disorders of pregnancy [odds ratio (OR) 1.170, 95% confidence interval (CI) 1.051–1.302, p = 0.004], in particular gestational hypertension (OR 1.083, 95% CI 1.007–1.164, p = 0.031), but not other pregnancy and perinatal diseases (all *p* > 0.05). Sensitivity analyses demonstrated pleiotropy only in pre-eclampsia or eclampsia (*p* = 0.0004), but not in other pregnancy and perinatal diseases (all *p* > 0.05). The results remained consistent after excluding two outliers (all *p* > 0.05).

**Conclusions:**

We confirmed a causal relationship between PCOS and hypertensive disorders of pregnancy, in particular gestational hypertension, but no association with any other adverse pregnancy or perinatal outcome. Therefore, we suggest that women with PCOS who are pregnant should have their blood pressure closely monitored.

## 1 Introduction

Polycystic ovary syndrome (PCOS) affects 10%–13% of reproductive-age women. It is characterized by anovulation, amenorrhea, hyperandrogenism, and polycystic ovary morphology (PCOM) (1). The pathophysiology of PCOS was associated with metabolic disorders, such as insulin resistance (IR), and endocrine-reproductive comorbidities (2), such as infertility, obesity, hirsutism, and cardiovascular problems (3). Women with PCOS often experience hyperandrogenism and IR, which have been associated with an increased risk of sporadic miscarriage and unfavorable obstetric outcomes during pregnancy (4). It has been well understood that the etiology of PCOS is the complex interplay of polygenetic and environmental elements (5). Previous reports have suggested that women with PCOS have an increased risk of maternal and fetal complications during pregnancy (4, 6–8).

Women with PCOS have reduced fertility potential, such as altered oocyte and endometrial competence and impaired endometrial–embryo cross-talk (9). In recent years, the reproductive outcomes of PCOS have become a research hotspot. Observational studies and meta-analyses have reported the relationship between PCOS and adverse pregnancy and perinatal outcomes (4, 6–8). It has been suggested that women with PCOS were at increased risk for miscarriage, gestational diabetes mellitus (GDM), gestational hypertension, and pre-eclampsia (4). A retrospective cohort study discovered that women with PCOS were more likely to experience preterm premature rupture of membrane (PPROM), preterm delivery, and placental abruption (8). However, the consensus on these effects is lacking. Cofactors related to PCOS, such as obesity, IR, glucose metabolism impairment, and metabolic syndrome, could influence endometrial competence, trophoblast invasion, placentation, pregnancy outcome, and even obstetric complications (9). Thus, the relationship between PCOS and pregnancy outcomes remains controversial because of confounding bias and methodological flaws in previous studies.

A Mendelian randomization (MR) study can estimate the causality between the exposure and outcome using instrumental variables (IVs) for the exposure and outcome. This method offers the advantage of reducing reverse causality and eliminating confounder bias (10, 11). In our MR study, the two‐sample MR approach can be more efficient and powerful for exploring the “gene‐risk factor” and “gene‐outcome” relationship from two independent groups in the same ancestry compared to the one‐sample MR approach (12). It was, therefore, useful to explain the relationship between PCOS and adverse pregnancy and perinatal outcomes in the genetic role (13).

The purpose of our study was to systematically investigate the causal effect of PCOS on adverse pregnancy and perinatal outcomes by conducting a two-sample MR analysis.

## 2 Material and methods

**Figure 1:**
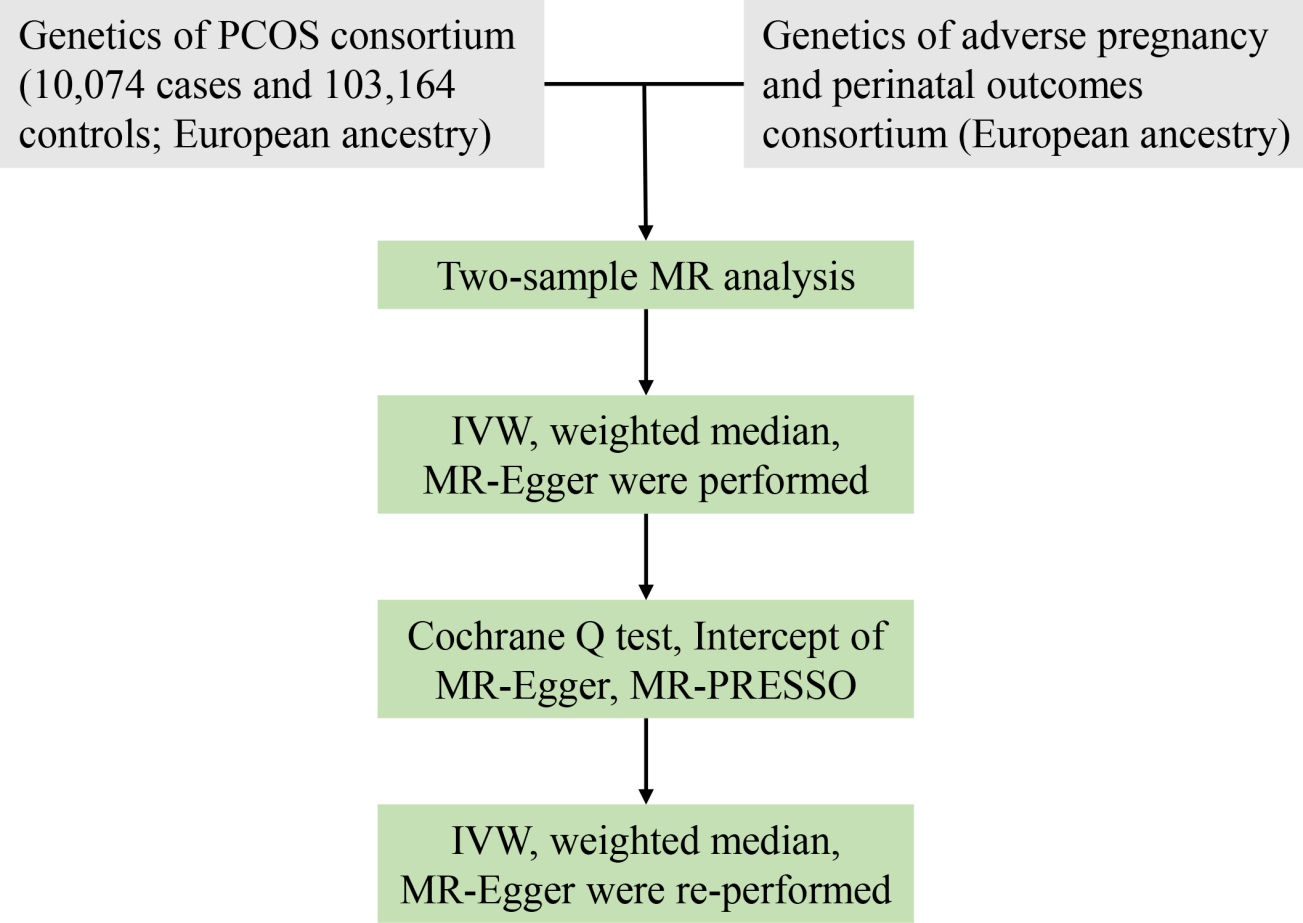
Workflow of MR study revealing causality from PCOS on adverse pregnancy and perinatal outcomes. PCOS, polycystic ovary syndrome; IVW, inverse variance weighted; MR, Mendelian randomization; MR-PRESSO, MR-pleiotropy residual sum and outlier; SNPs, single-nucleotide polymorphisms.

To evaluate the causative influence of PCOS on adverse pregnancy and perinatal outcomes, we conducted a two-sample MR study. Published genome-wide association study (GWAS) meta-analyses (14–16) provided the pooled data. **Figure 1** illustrates the overview of the research design.

### 2.1 GWAS data for PCOS

Day et al. (14, 15) performed the biggest GWAS meta-analysis of PCOS in European ancestry, with 10,074 cases and 103,164 controls (**Supplementary Table S1**). The diagnosis of PCOS was according to the Rotterdam criteria (17), National Institutes of Health criteria (NIH/NICHD) (18), or self-report questionnaire (19). NIH/NICHD criteria were satisfied by the presentation of both hyperandrogenism, such as hirsutism or acne, and ovulatory dysfunction, such as oligomenorrhea or amenorrhea, whereas the Rotterdam criteria required two out of three major features to be presented and the existence of PCOM. In the 23andMe (Mountain View, CA, USA) cohort, the self-reported diagnosis was employed; however, summary-level data from 4,890 cases and 20,405 controls included in this cohort were not available because of the data sharing policy. The GWAS meta-analysis elucidated shared genetic structure across the three diagnostic criteria (14).

### 2.2 IV selection

The instruments chosen for exposure (PCOS) had to satisfy the following criteria to ensure the validity of the IVs included in our MR study: single-nucleotide polymorphisms (SNPs) were associated with exposure at the threshold of genome-wide significance (p < 5 × 10^−8^) (20), all SNPs should follow the linkage equilibrium (pairwise r^2^ ≤ 0.01 in the current study), and *F* statistic above 10 was required for sufficient strength to limit the bias from weak IVs (21). We used *R* ^2^ × (N − k − 1)/[(1 − *R* ^2^) × k] to calculate the *F* statistic, where N means the sample size of GWAS, k refers to the number of SNPs, and *R* ^2^ is the ratio of the variability of PCOS explained by each SNP. Specifically, *R* ^2^ is calculated using the formula [2 × β^2^ × (1 − EAF) × EAF]/[2 × β^2^ × (1 − EAF) × EAF + 2 × N × SE^2^ × (1 − EAF) × EAF], where EAF is the effect allele frequency, β is the estimate of the genetic effect of each SNP on PCOS, and SE is the standard error of beta (21). **Supplementary Table S2** shows detailed genetic information on selected SNPs. SNPs linked to exposure were retrieved from outcome data (adverse pregnancy and perinatal outcomes). To reduce the possible bias from population heterogeneity, all the GWAS consortia employed in our MR study were restricted to those of European ancestry.

### 2.3 GWAS data for adverse pregnancy and perinatal outcomes

We examined associations with 14 outcomes: sporadic miscarriage, GDM, hypertensive disorders of pregnancy, gestational hypertension, pre-eclampsia or eclampsia, polyhydramnios, intrahepatic cholestasis of pregnancy (ICP), placenta disorder, placental abruption, placenta previa, premature rupture of membranes (PROM), postpartum hemorrhage, postpartum depression, and poor fetal growth. The definitions of these outcomes in FinnGen (16) are provided in **Supplementary Table S1**. The FinnGen study is a countrywide Finnish GWAS meta-analysis that includes nine biobanks and has minimal overlap with the PCOS GWAS, thereby reducing the potential bias arising from overlapping samples (22). FinnGen includes sporadic miscarriage (*N* = 15073 cases/135,962 controls), GDM (*N* = 11,279 cases/179,600 controls), hypertensive disorder of pregnancy (*N* = 13,071 cases/177,808 controls), gestational hypertension (*N* = 7,503 cases/176,113 controls), pre-eclampsia or eclampsia (*N* = 6,436 cases/176,113 controls), polyhydramnios (*N* = 1,049 cases/154,102 controls), ICP (*N* = 2,196 cases/188,683 controls), placenta disorder (*N* = 193 cases/154,102 controls), placenta previa (*N* = 1,076 cases/154,102 controls), placental abruption (*N* = 546 cases/154,102 controls), PROM (*N* = 6,129 cases/154,102 controls), postpartum hemorrhage (*N* = 7,221 cases/148,153 controls), postpartum depression (*N* = 13,657 cases/236,178 controls), and poor fetal growth (*N* = 3,056 cases/187,823 controls), and those outcomes were defined based on International Classification of Diseases (ICD) codes (16). In addition, hypertensive disorders of pregnancy encompass gestational hypertension, pre-eclampsia or eclampsia, chronic hypertension, and chronic hypertension with superimposed pre-eclampsia.

### 2.4 MR estimates

From the GWAS meta-analysis of the outcome, we retrieved and extracted IVs for PCOS. We ruled out SNPs linked to outcome (adverse pregnancy and perinatal outcomes) (*p* < *5* × 10^−8^) or absent in the outcome data pool. We harmonized the effect alleles across the GWASs of PCOS and pregnancy outcomes and then excluded those that were palindromic based on the information of EAF (default EAF > 0.42 of the “harmonisation” function in the “Two-Sample MR” package). We employed the inverse variance weighted (IVW) method as the major of MR estimation to examine the causality of PCOS on the risk of pregnancy outcomes. Based on the MR assumptions, this method supposed that all IVs were effective and combined the Wald ratio estimates of the causal effect by different SNPs to offer an identical assessment of the causal effect of PCOS on the pregnancy outcomes (12). Then, we obtained a *post-hoc* power calculation through the IVW model (https://shiny.cnsgenomics.com/mRnd/) (23).

### 2.5 Sensitivity analyses

In MR studies, sensitivity analysis has been proven crucial in detecting the pleiotropy and heterogeneity for MR estimations that may significantly violate the MR assumptions. We used Cochran’s Q test to characterize potential heterogeneity derived from the IVW approach. The directional pleiotropy was shown by the intercept achieved from MR-Egger regression (*p* < 0.05 referred to as the existence of directional pleiotropy) (24). In addition, it is universal to employ MR-pleiotropy residual sum and outlier (MR-PRESSO) methods to evaluate and correct horizontal pleiotropy (25). MR-PRESSO included the following three contents: a) testing of significant results in the causal estimates before and after correction for outliers, b) correction for horizontal pleiotropy through outlier removal, and c) detection of horizontal pleiotropy. When the condition of parallel pleiotropy variants’ percentage is <10%, it minimizes bias and has greater precision than IVW and MR-Egger (25). Moreover, we performed leave-one-out analyses to assess whether a single SNP could drive and influence the MR estimate.

The “Two-Sample MR” package (version 0.5.6) and “MR-PRESSO” package (version 1.0) were used to conduct all of the analyses in the R program (version 3.6.1). Results with *p*-value <0.05 were considered to be significant.

## 3 Results

The study includes 14 PCOS-related SNPs that met the threshold of genome-wide significance with LD r^2^ ≤ 0.01. However, two SNPs (rs11225154 and rs853854) were not directly matched in the outcome data and were therefore not used in further analysis. After ruling out SNPs significantly linked to adverse pregnancy and perinatal outcomes (*p* < 5 × 10^−8^), the remaining SNPs were used for analysis in our study. Only one excluded SNP (rs7563201) was strongly linked to gestational hypertension.

**Figure 2:**
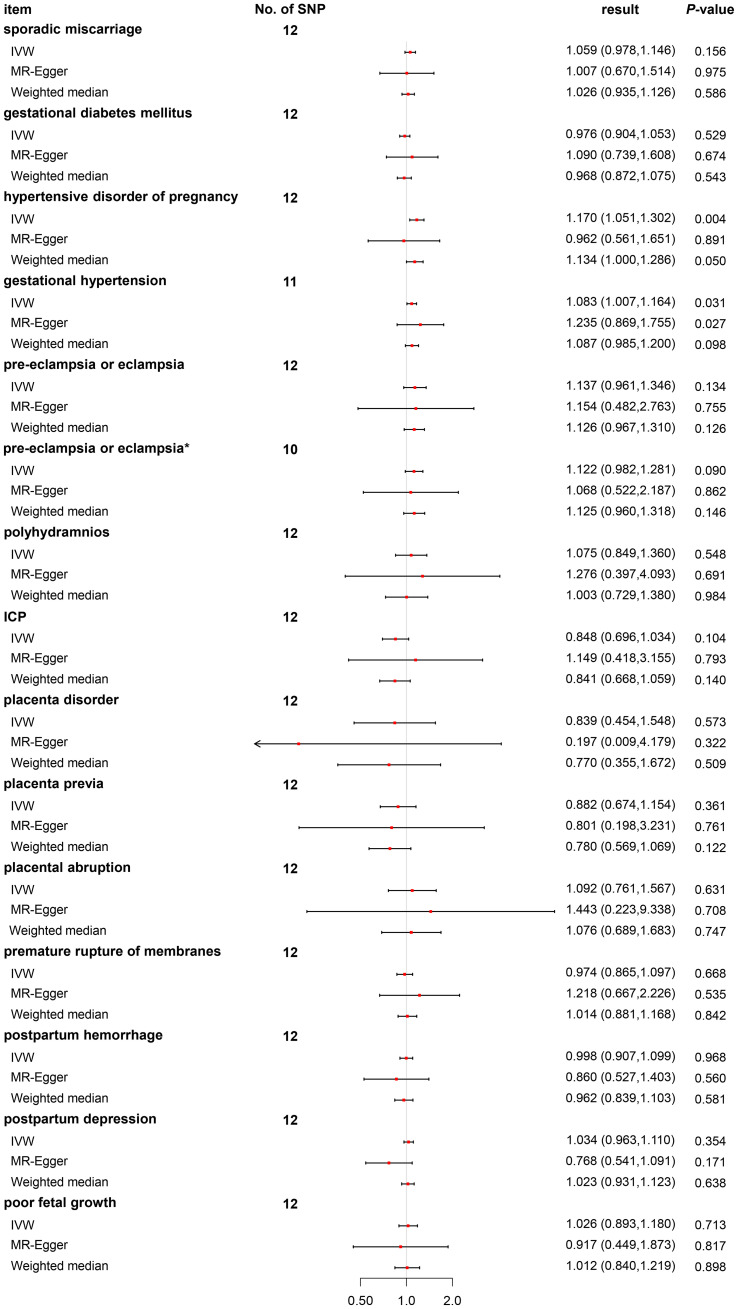
Odds ratio plot for PCOS and adverse pregnancy and perinatal outcomes. OR, odds ratio; PCOS, polycystic ovary syndrome. *Two outliers (rs11225154 and rs853854) were weeded out.

We discovered no relationships between the causal effect of PCOS and sporadic miscarriage [odds ratio (OR) 1.059, 95% CI 0.978–1.146, *p* = 0.156], GDM (OR 0.976, 95% CI 0.904–1.053, *p* = 0.529), pre-eclampsia or eclampsia (OR 1.137, 95% CI 0.961–1.346, *p* = 0.134), polyhydramnios (OR 1.075, 95% CI 0.849–1.360, *p* = 0.548), ICP (OR 0.848, 95% CI 0.696–1.034, *p* = 0.104), placenta disorder (OR 0.839, 95% CI 0.454–1.548, *p* = 0.573), placenta previa (OR 0.882, 95% CI 0.674–1.154, *p* = 0.361), placenta abruption (OR 1.092, 95% CI 0.761–1.567, *p* = 0.631), PROM (OR 0.974, 95% CI 0.865–1.097, *p* = 0.668), postpartum hemorrhage (OR 0.998, 95% CI 0.907–1.099, *p* = 0.968), postpartum depression (OR 1.034, 95% CI 0.963–1.110, *p* = 0.354), and poor fetal growth (OR 1.026, 95% CI 0.893–1.180, *p* = 0.713) by the IVW method (as shown in **Figure 2**). *Post-hoc* analyses revealed a power of 0.009–0.700 for the IVW model (**Table 1**).

**Table 1:** MR results of heterogeneity and directional pleiotropy.

Item	Power	Heterogeneity	Global heterogeneity test	Directional pleiotropy		
		*p-*Value	*p-*Value	Intercept	SE	*p-*Value
Sporadic miscarriage	0.380	0.138	0.155	0.006	0.026	0.810
GDM	0.090	0.416	0.416	−0.014	0.025	0.582
Hypertensive disorders of pregnancy	0.990	0.157	0.179	0.025	0.035	0.485
Gestational hypertension	0.370	0.468	0.477	−0.017	0.023	0.472
Pre-eclampsia or eclampsia	0.700	**0.0004**	**0.001**	−0.002	0.056	0.975
Pre-eclampsia or eclampsia*	/	0.107	0.112	0.007	0.048	0.894
Polyhydramnios	0.009	0.587	0.616	−0.022	0.748	0.775
ICP	0.380	0.135	0.144	−0.039	0.065	0.562
Placenta disorder	0.080	0.243	0.256	0.186	0.196	0.365
Placenta previa	0.150	0.199	0.224	0.012	0.089	0.892
Placenta abruption	0.080	0.260	0.281	−0.036	0.120	0.771
PROM	0.080	0.163	0.167	−0.029	0.039	0.475
Postpartum hemorrhage	0.050	0.358	0.363	0.019	0.031	0.557
Postpartum depression	0.140	0.741	0.759	0.038	0.022	0.121
Poor fetal growth	0.060	0.453	0.457	0.014	0.046	0.758

GDM, gestational diabetes mellitus; ICP, intrahepatic cholestasis of pregnancy; PROM, premature rupture of membranes; MR, Mendelian randomization. Bold text indicates statistical significance (p<0.05). "/" indicates that it is not calculated.

*Two outliers (rs11225154 and rs853854) were weeded out.

We found a causal relationship between PCOS and hypertensive disorders of pregnancy (OR 1.170, 95% CI 1.051–1.302, *p* = 0.004) by the IVW method (as shown in **Figure 2**). As hypertensive disorders of pregnancy have several subtypes, further analyses revealed only causal effects of PCOS and gestational hypertension (OR 1.083, 95% CI 1.007–1.164, *p* = 0.031), but not pre-eclampsia or eclampsia (OR 1.137, 95% CI 0.961–1.346, *p* = 0.134) (**Figure 2**). *Post-hoc* analyses revealed power of 0.990 and 0.370 for the IVW model (**Table 1**).

We performed a sensitivity analysis using MR-Egger regression and weighted mean approaches. For most outcomes, consistent magnitude and direction of MR estimates were obtained (**Figure 2**). Further, no significant heterogeneity was observed with *p*-value >0.05 of IVW by Cochran’s Q test, except for pre-eclampsia or eclampsia (*p* = 0.0004). The same conclusion was also gained using MR-PRESSO, with *p*-value >0.05, except for pre-eclampsia or eclampsia (*p* = 0.001) (**Table 1**). In addition, no evidence showed a significant intercept (*p* > 0.05), suggesting that no directional pleiotropy was observed. Some single SNPs affected the overall effect of PCOS on adverse pregnancy and perinatal outcomes in the leave-one-out sensitivity analysis (**Supplementary Figure S1**).

For pre-eclampsia or eclampsia, heterogeneity was also investigated using a standard Cochran’s Q test, which derived a *p*-value <0.001 of IVW. MR-PRESSO also presented a similar result (global heterogeneity test *p* = 0.001). After weeding out two outliers (rs2271194 and rs7563201), the same MR approach followed by the IVW method was conducted again. As expected, further results demonstrated that the result was consistent with the previous (before correction, OR 1.137, 95% CI 0.961–1.346, *p* = 0.134 *vs*. after correction, OR 1.122, 95% CI 0.982–1.281, *p* = 0.090) (**Figure 2**).

## 4 Discussion

### 4.1 Principal findings

In the present study, a two-sample MR method was applied to assess whether PCOS adversely influenced pregnancy and perinatal outcomes in a causal effect. Our results showed that PCOS played a confirmative genetic role in the risk of hypertensive disorders of pregnancy, in particular gestational hypertension, but not pre-eclampsia or eclampsia.

### 4.2 Results in the context of what is known

PCOS has multiple etiologies associated with various genetic and environmental factors (1). It has many metabolic symptoms, such as central obesity, hyperandrogenism, elevated fasting blood glucose, and IR. PCOS and its comorbidities are linked to altered endometrial competence, oocyte quality, and impaired endometrial–embryo cross-talk, which increase the risk of infertility and early or late obstetric complications through abnormal trophoblast invasion and placentation (9). In addition, maternal exposure to 5α-dihydrotestosterone (DHT) and IR in a PCOS rat model changed the ferroptosis pathway in the gestational uterus and placenta, which was associated with increased necroptosis in the placenta and reduced the activation of apoptosis in the uterus, leading to miscarriage (26).

Reproductive outcome is one of the most essential concerns for women with PCOS in childbearing age. Therefore, in clinical studies, investigating the relationship between PCOS and adverse pregnancy and perinatal outcomes is necessary, but up to now, it has remained unclear (4, 6–8). The previous observational studies had the limitation of possible bias from confounding factors. However, adequately powered and well-designed cohort studies or prospective trials with long-term follow-up would be very costly in terms of time, money, labor, and material resources. Moreover, findings from observational studies have not been sufficient to draw conclusions on cause–effect relationships. Compared with previous methods, MR is more effective and practical to comprehensively reveal these causalities.

We discovered a higher risk of hypertensive disorders of pregnancy, consistent with previous results (27). Hypertensive disorders of pregnancy encompass four subtypes. We tried to clarify which subtype was most likely to be affected. We discovered that PCOS only exerted causal effects on the risk of gestational hypertension (**Figure 2**), but not pre-eclampsia or eclampsia (**Figure 2**), chronic hypertension (**Supplementary Table S3**), and chronic hypertension with superimposed pre-eclampsia (**Supplementary Table S3**). Possibly, it was that just gestational hypertension derived the causal relationship between PCOS and hypertensive disorders of pregnancy.

Several systematic reviews have summarized previous studies and come to different conclusions; nonetheless, the results of those pooled analyses suggested that women with PCOS were at increased risk of hypertensive disorders of pregnancy and pre-eclampsia (4, 6–8). Hyperinsulinemia and IR exacerbated endothelial injury and interfered with endothelium-dependent vasodilation, resulting in dyslipidemia and muscular hypertrophy of the vascular wall. High levels of free testosterone induced sympathetic and vascular hyper-responsiveness, both of which in PCOS were important for the occurrence and development of hypertensive disorders of pregnancy (28). Rs7563201, as one of the IVs of PCOS in our MR study, was associated with the expression of *THADA* (https://www.ncbi.nlm.nih.gov/snp/rs7563201). *THADA* was shown to have metabolic contributions to the pathophysiology of PCOS, such as disorders of glucose metabolism, hyperandrogenism, and dyslipidemia (29), which could also contribute to hypertensive disorders of pregnancy (28).

Possible factors were considered regarding our negative findings. First, the effect of PCOS on adverse pregnancy and perinatal outcomes was slightly lower than expected. In conventional regression analysis, we may ignore the bias from reverse causation or common risk factors. Second, vertical pleiotropy may exert efforts. Hyperandrogenism level and IR, which were genetically related, could lead to a more susceptible status in the evolution of PCOS. Thus, the detailed mechanisms underlying PCOS and pregnancy and perinatal outcomes were complicated and deserving of further investigation. Especially, as PCOS is a widely varying disease, the criteria for PCOS diagnosis should be restricted in future research.

### 4.3 Clinical implications

These findings suggested that PCOS was causally associated with hypertensive disorders of pregnancy, in particular gestational hypertension, which were among the idiopathic diseases of pregnancy, posing serious threats to the health of mothers and infants. It was suggested that the blood pressure of all pregnant women with PCOS should be closely monitored.

### 4.4 Strengths and limitations

Our study had several strengths. First, we effectively reduced the occurrence probability of reverse causality and confounding bias using the MR method, which genetically predicted phenotype as the exposure of interest. Second, the data we recruited were GWAS summary data, which came from the largest scale of recent meta-studies, which may, to a large extent, reduce the bias related to population heterogeneity in European people.

However, the study also had some limitations. First, GWAS data utilized in our study came from a European population. For this reason, this kind of relationship needs to be confirmed in demographically different populations such as Asian individuals. Second, because there were three main diagnostic criteria of PCOS set by the NIH/NICHD (18), Rotterdam criteria (17), and the Androgen Excess and PCOS Society (30), we could not distinguish what kind of phenotypes were more influential. The Rotterdam criteria described four symptoms of PCOS, and there were differences in hormones and metabolism between these groups (11). Furthermore, since the associations between PCOS phenotype and adverse pregnancy and perinatal outcomes were untested, the manifestations of PCOS may present with variety, indicating that the effects from specific characteristics of PCOS subgroups may be ignored or defaulted. A third limitation was that we analyzed PCOS as a binary risk factor. However, the development of PCOS was progressive and successive. The *post-hoc* powers were low in many outcomes. Therefore, it was difficult to interpret our obtained effect estimate, as our included genetic variants did not represent all risks of different subtypes of PCOS. The MR study for a relationship between PCOS and adverse pregnancy and perinatal outcomes was still valid (31).

## 5 Conclusion

In this study, using MR analysis, we demonstrated a significant effect between PCOS and hypertensive disorders of pregnancy, in particular gestational hypertension, but found no association with any other adverse pregnancy or perinatal outcome. Therefore, we suggest that women with PCOS who are pregnant should have their blood pressure closely monitored.

## Data availability statement

The datasets presented in this study can be found in online repositories. The names of the repository/repositories and accession number(s) can be found in the article/**Supplementary Material**.

## Ethics statement

Ethical approval was not required for the study involving humans in accordance with the local legislation and institutional requirements. Approval by a formal institutional review board was not required because this analysis consisted of a collection of published studies. Written informed consent to participate in this study was not required from the participants or the participants' legal guardians/next of kin in accordance with the national legislation and the institutional requirements.

## Author contributions

YM: Writing – review & editing, Writing – original draft. JC: Writing – original draft. LL: Writing – original draft, Writing – review & editing. TW: Writing – review & editing. WNH: Writing – original draft. WHH: Writing – review & editing. ZW: Writing – review & editing. YX: Writing – review & editing. YWX: Writing – review & editing. YW: Writing – review & editing. QM: Writing – review & editing.

## Supplementary material

Supplementary Figure 1 MR results of leave-one-out sensitivity analysis.

Supplementary Table 1 Details of the GWAS included in the MR.

Supplementary Table 2 Details of instrumental variables included in the MR.

Supplementary Table 3 MR results of chronic hypertension and chronic hypertension with superimposed pre-eclampsia.
