# Supplementary material for: Causal relationships exist between polycystic ovary syndrome and adverse pregnancy and perinatal outcomes: a Mendelian randomization study
Source: Front Endocrinol (Lausanne). 2024 Jun 28;15:1327849. doi: 10.3389/fendo.2024.1327849 (PMC11239544; doi:10.3389/fendo.2024.1327849)
Supplement: Supplementary Figure 1 — MR results of leave-one-out sensitivity analysis. [file Presentation_1.pptx]

## Slide 1
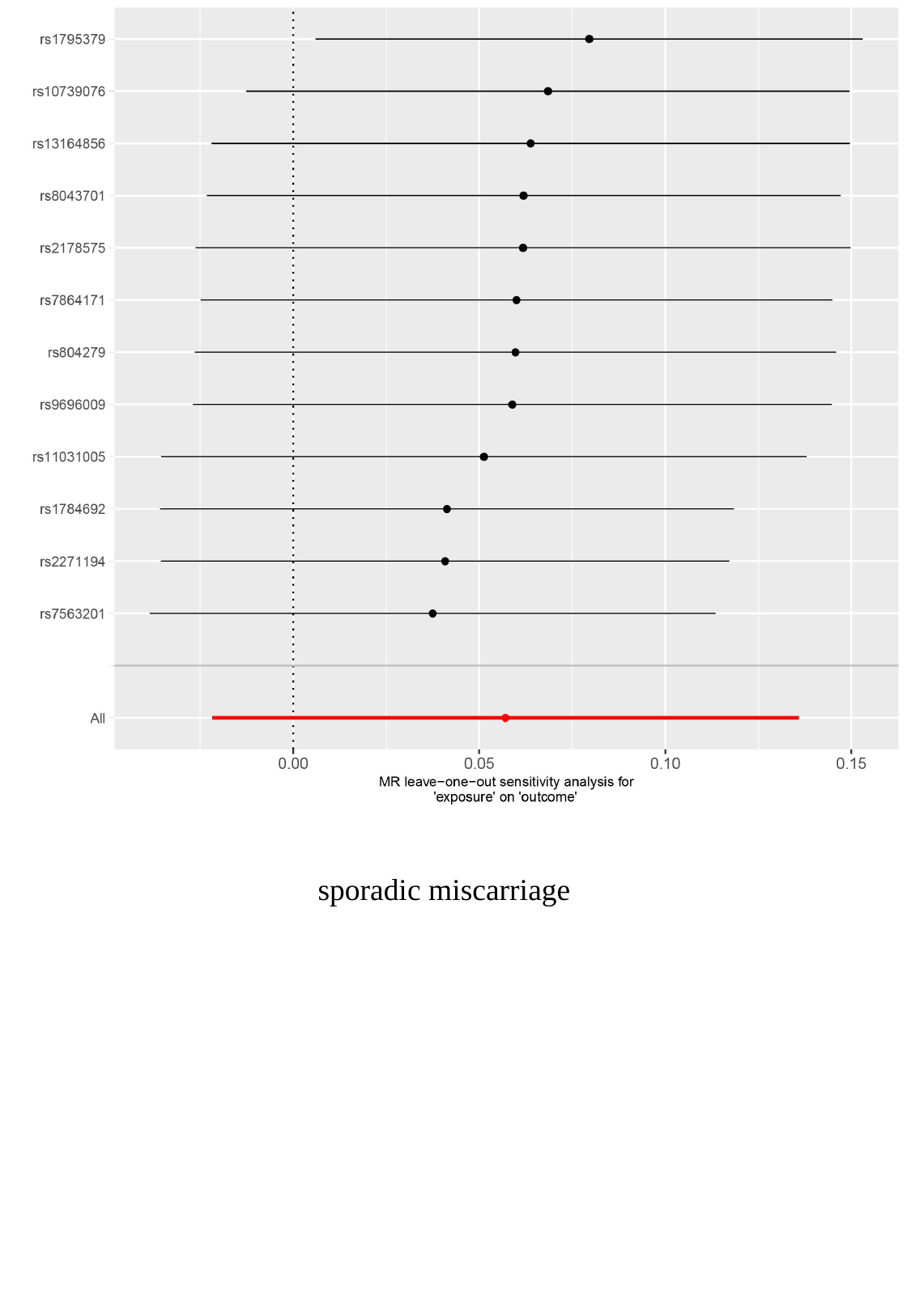

#
sporadic miscarriage

## Slide 2
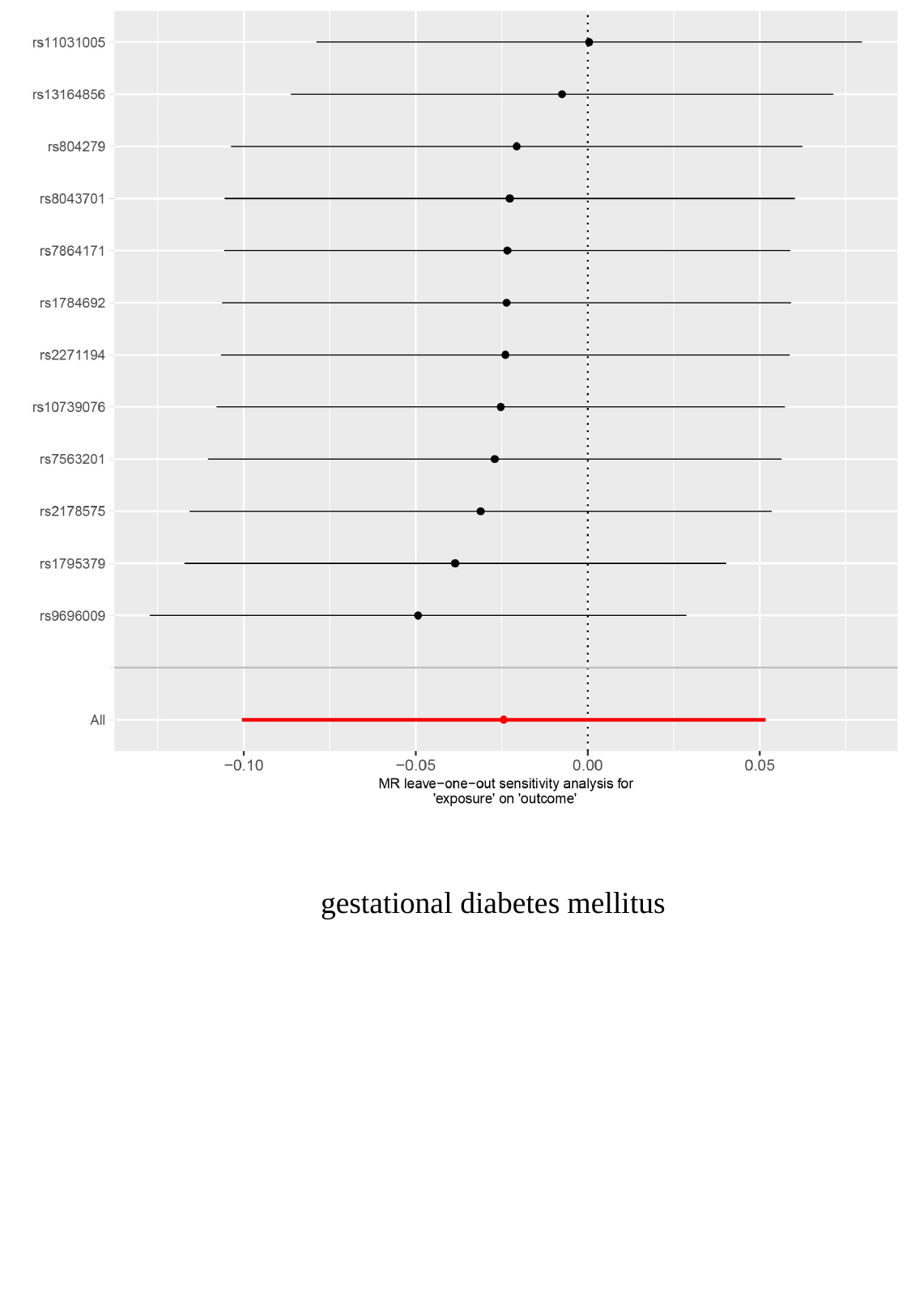

gestational diabetes mellitus

## Slide 3
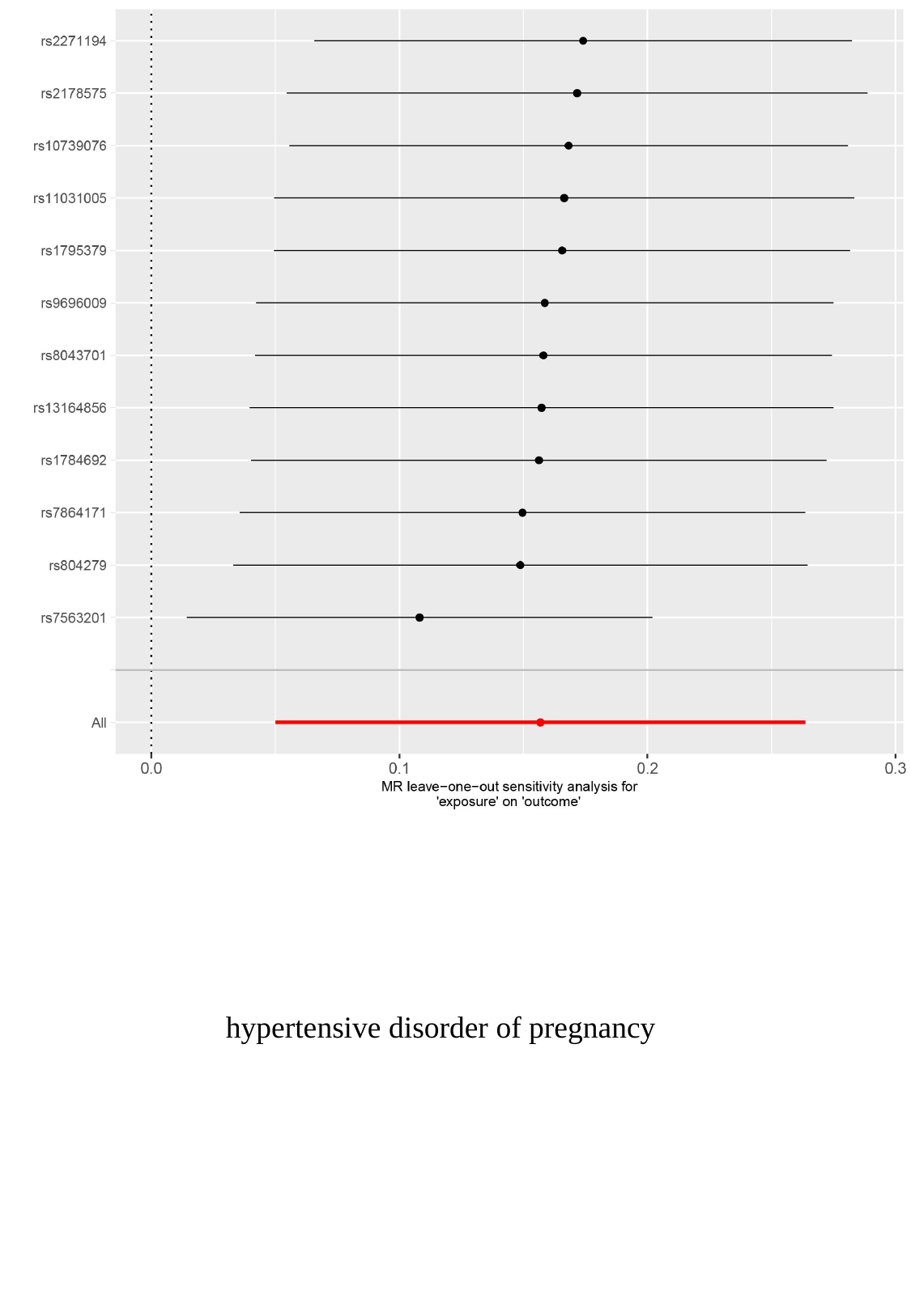

hypertensive disorder of pregnancy

## Slide 4
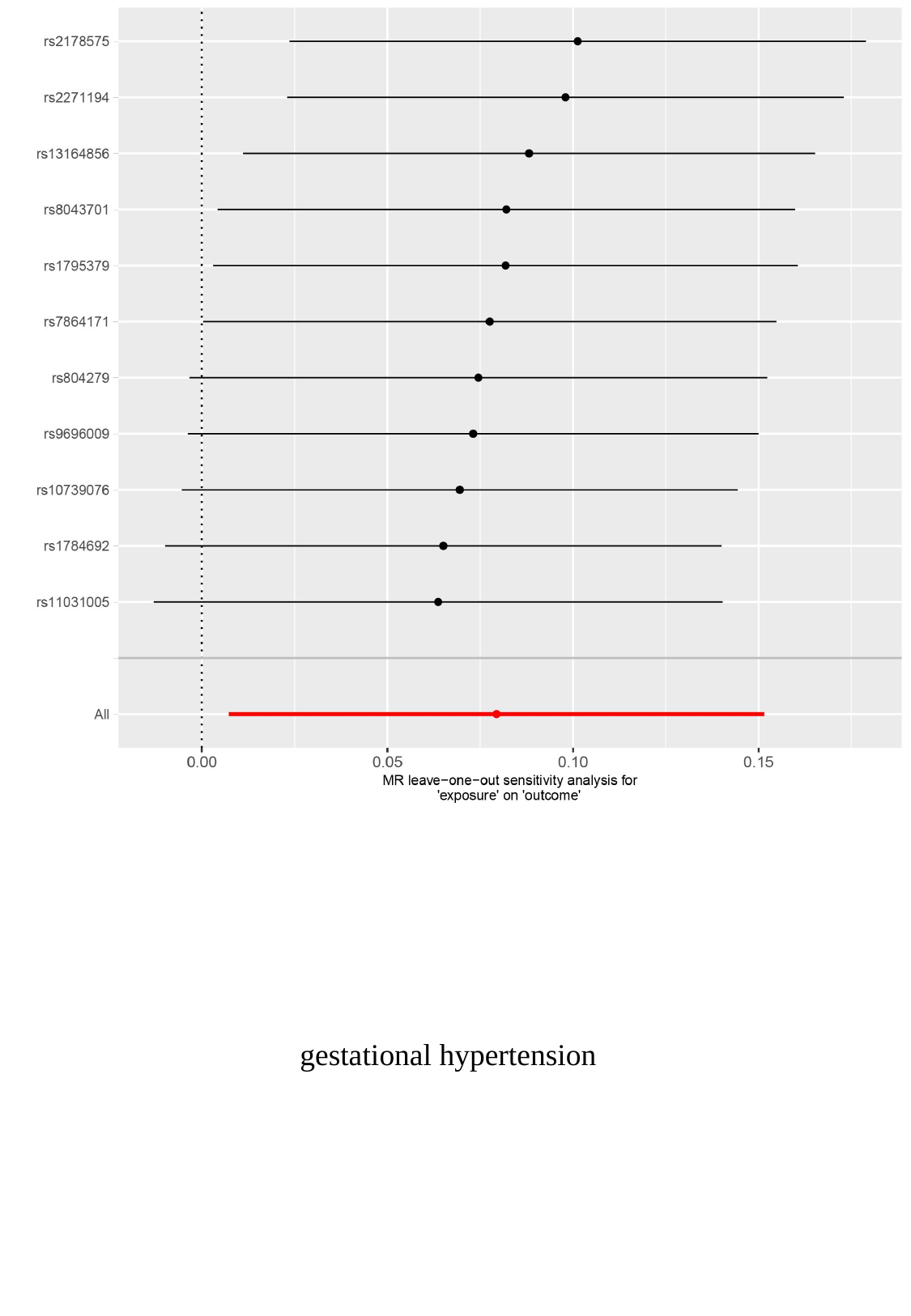

gestational hypertension

## Slide 5
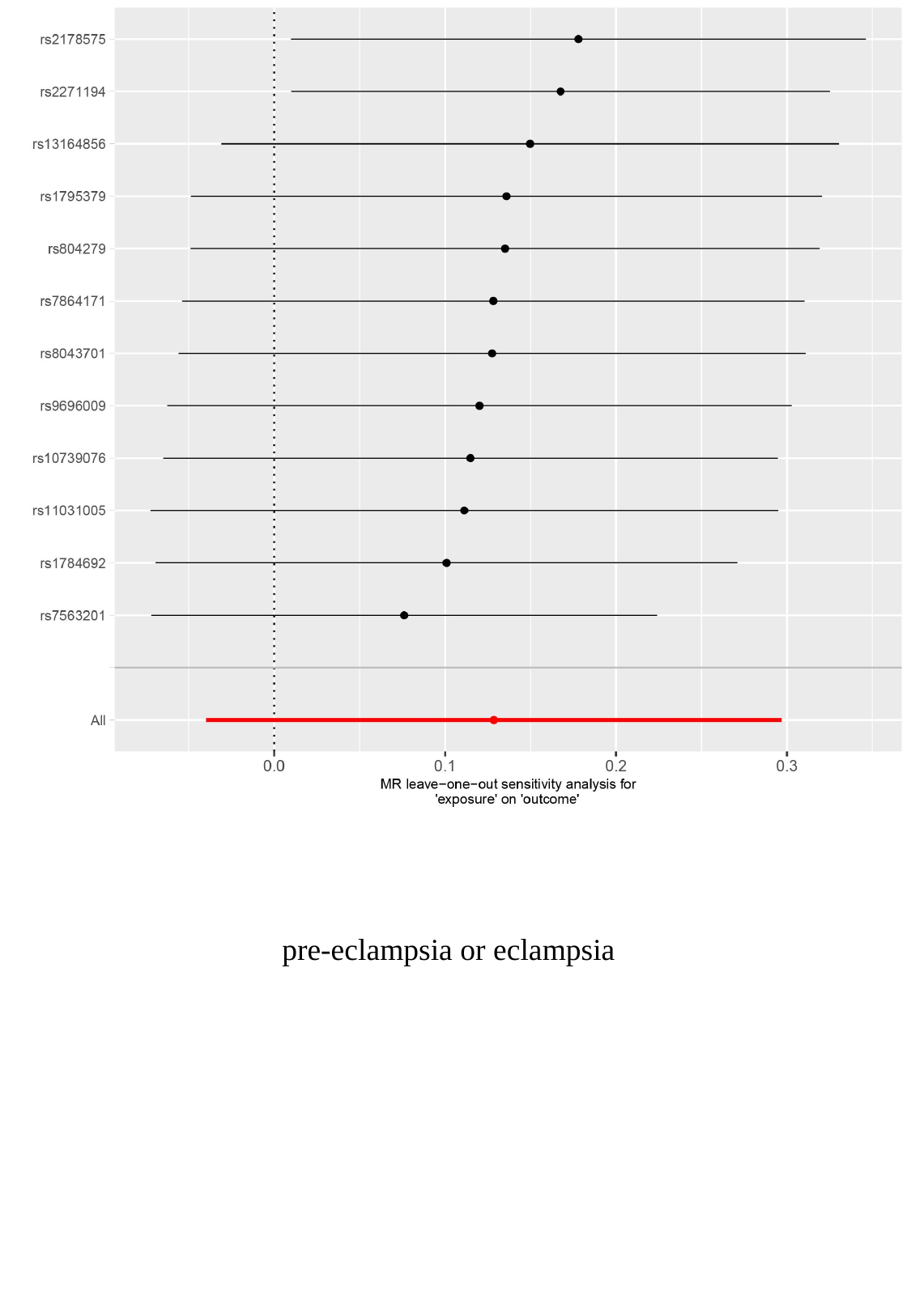

pre-eclampsia or eclampsia

## Slide 6
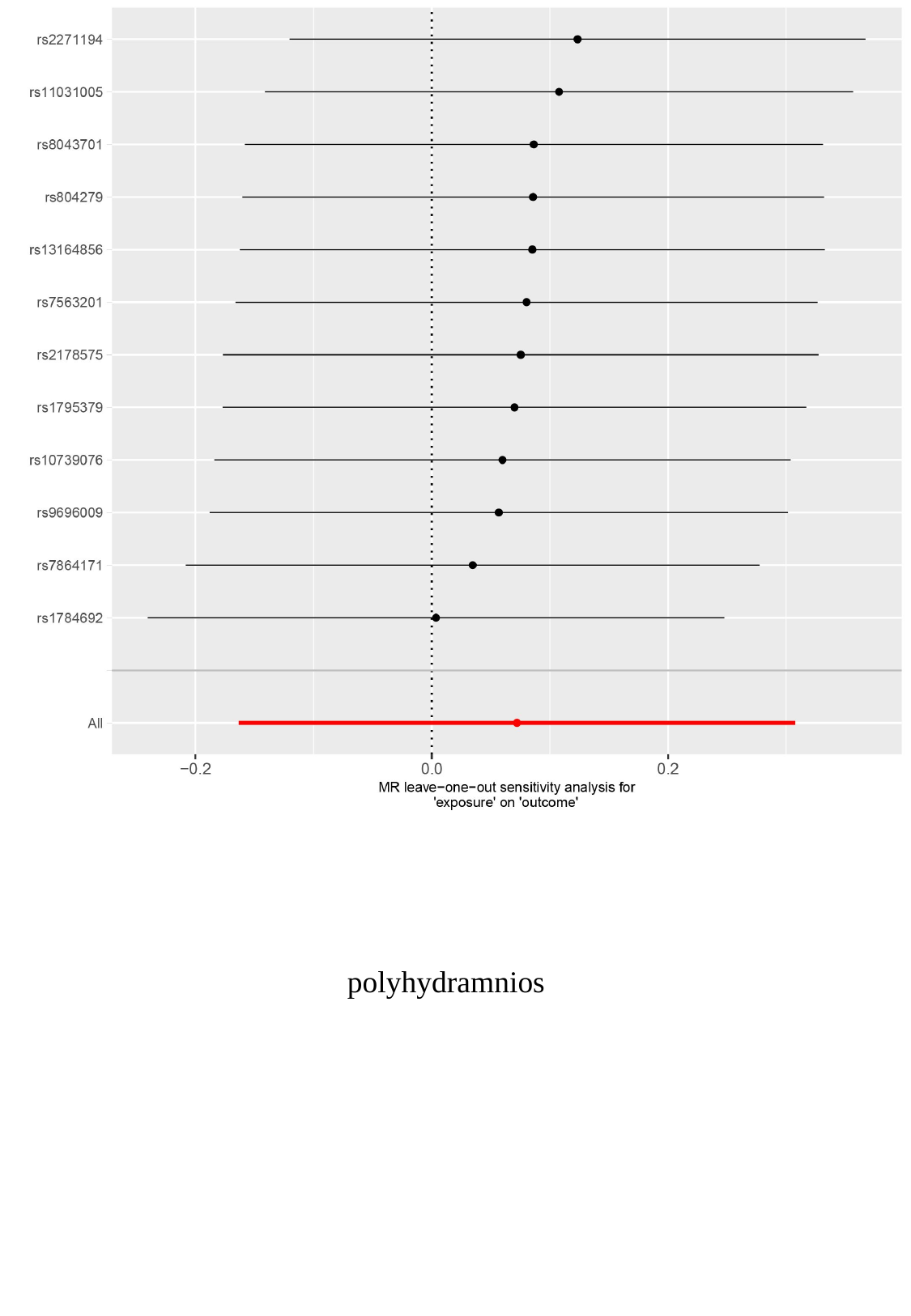

polyhydramnios

## Slide 7
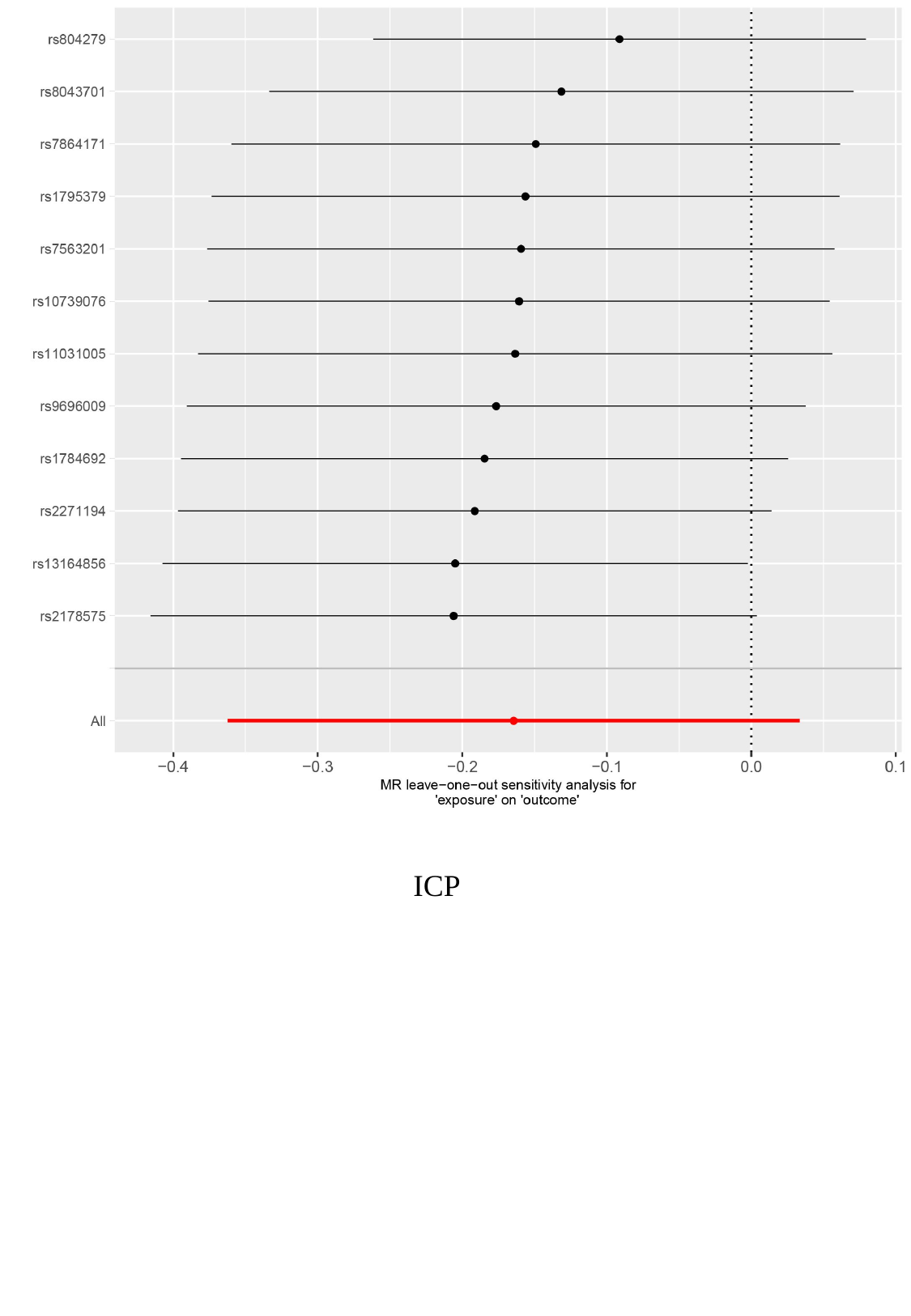

ICP

## Slide 8
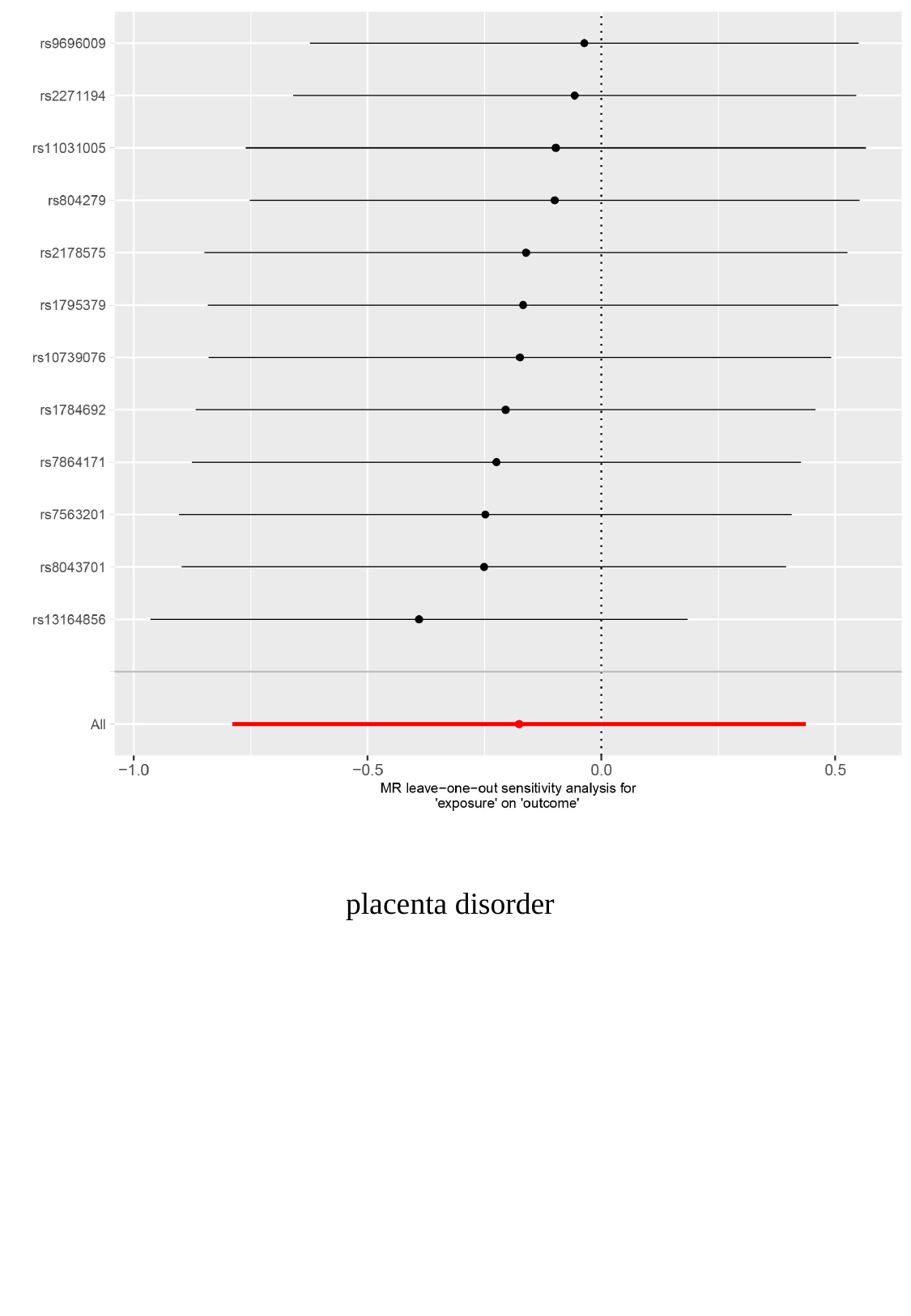

placenta disorder

## Slide 9
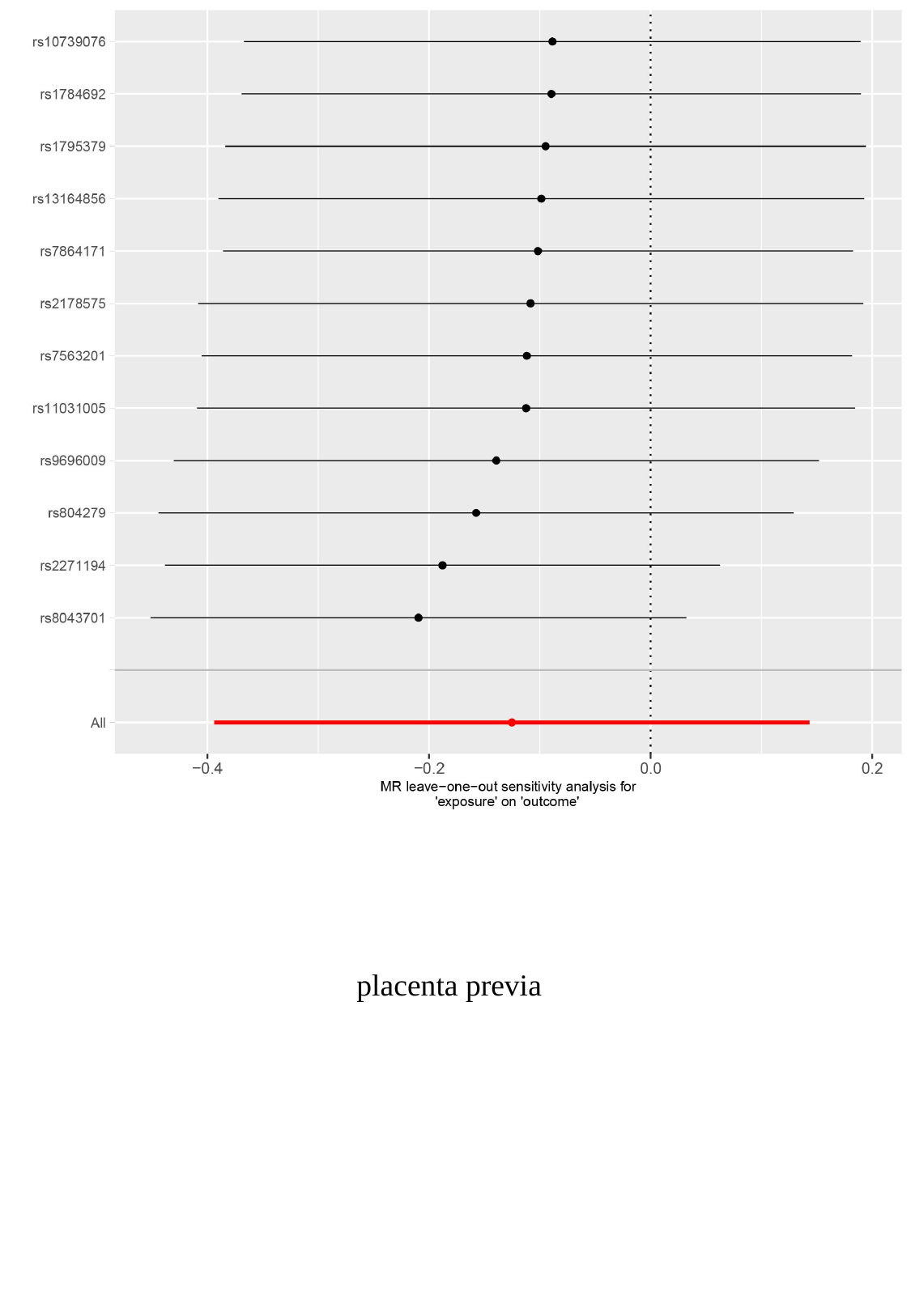

placenta previa

## Slide 10
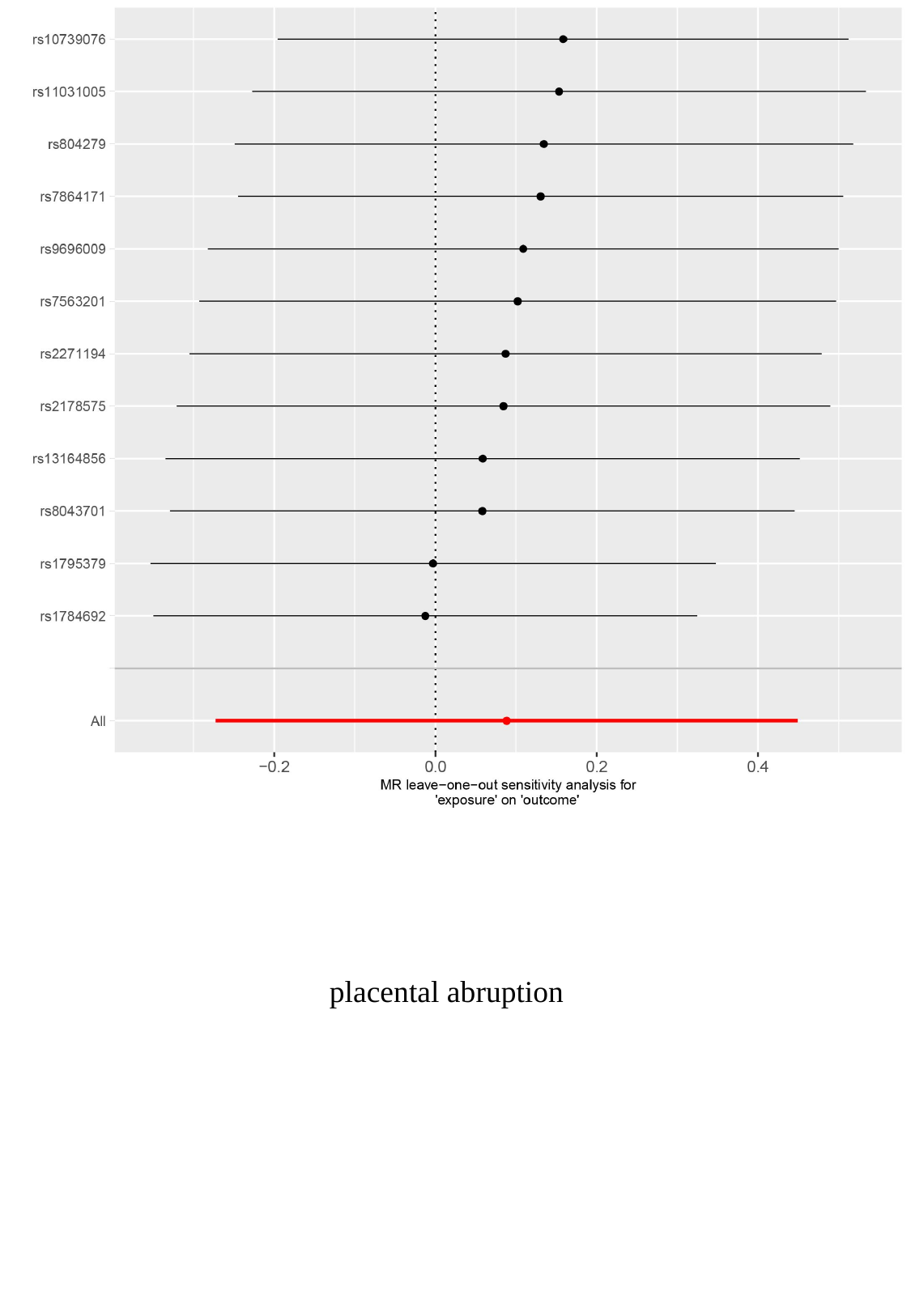

placental abruption

## Slide 11
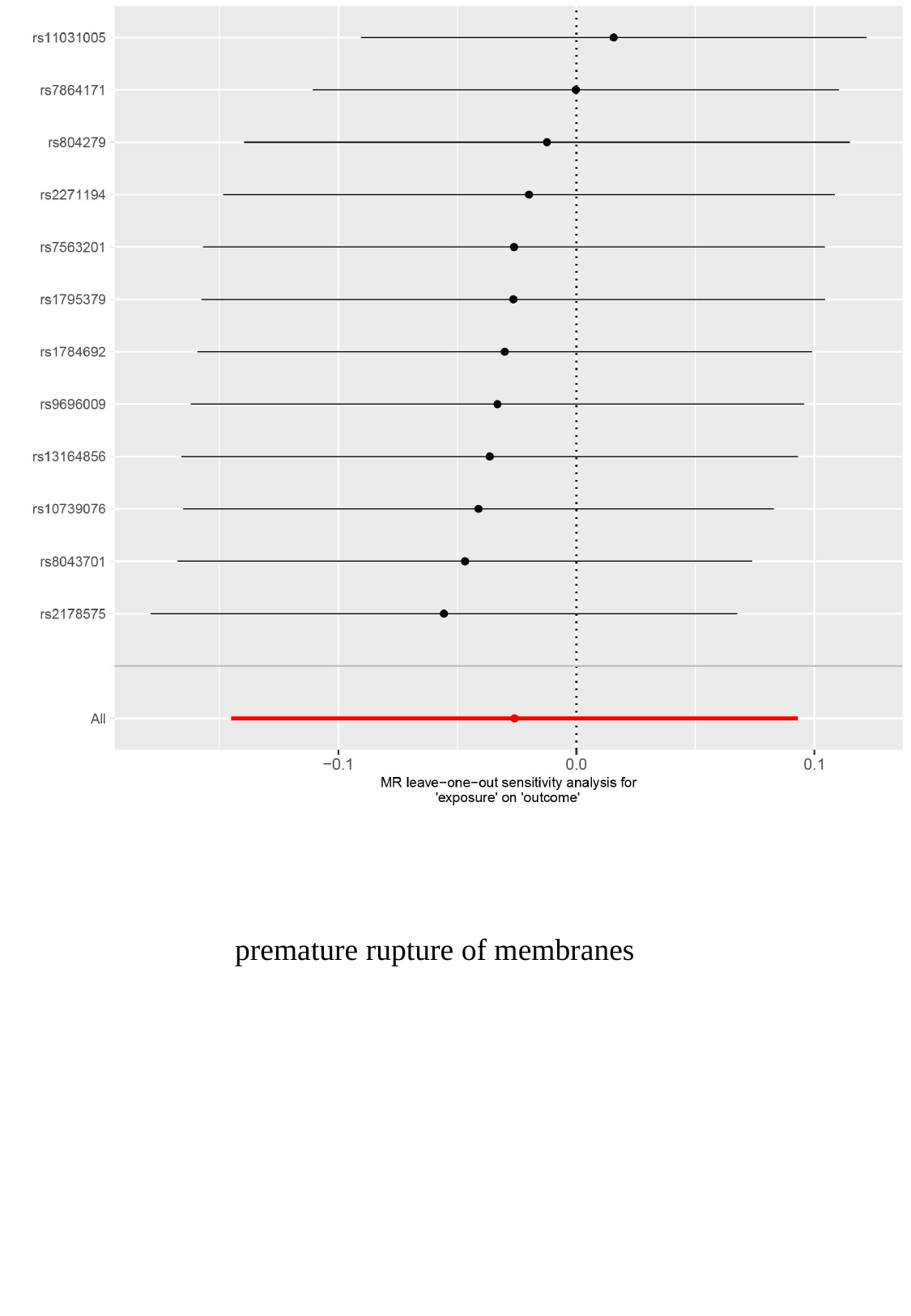

premature rupture of membranes

## Slide 12
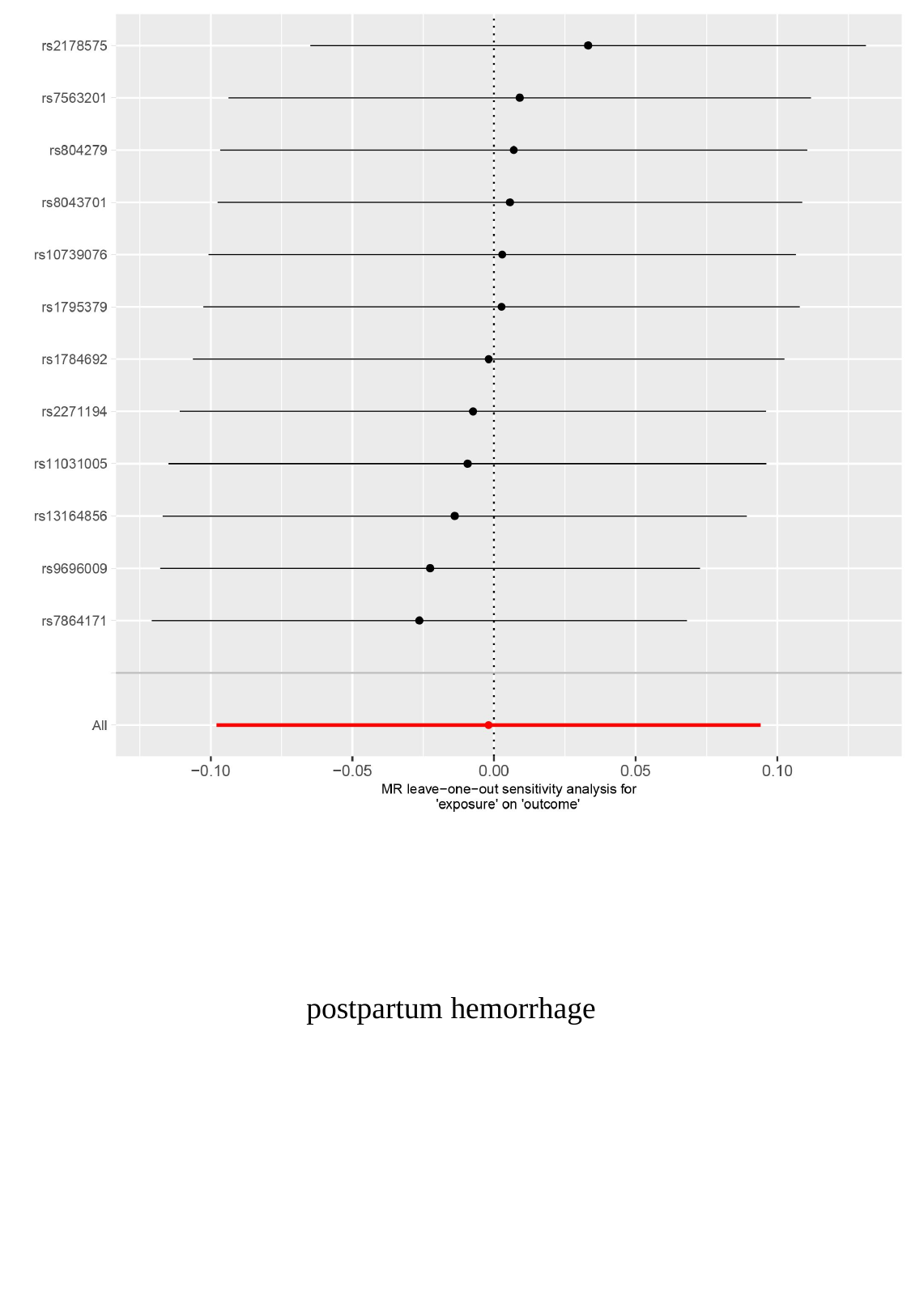

postpartum hemorrhage

## Slide 13
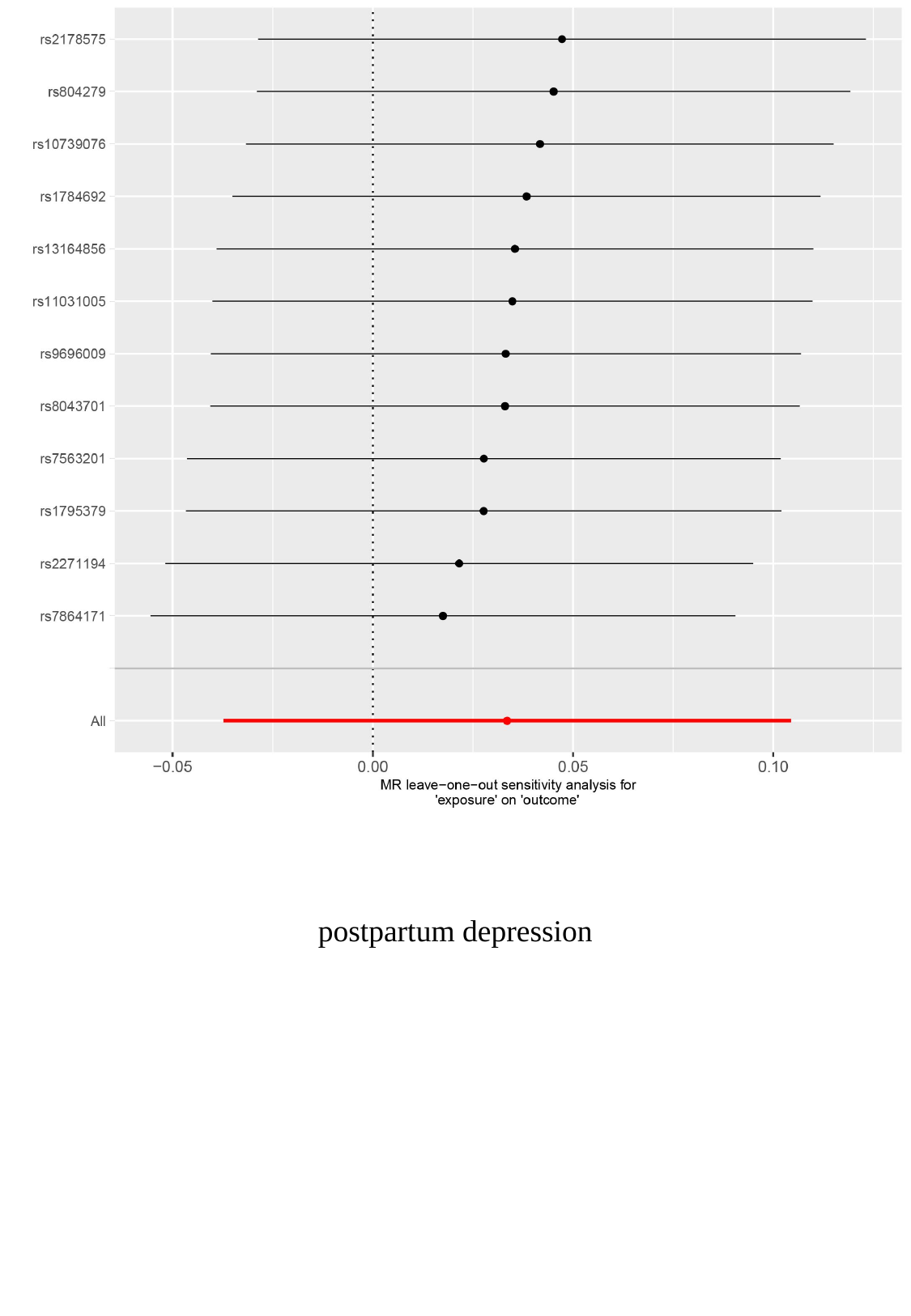

postpartum depression

## Slide 14
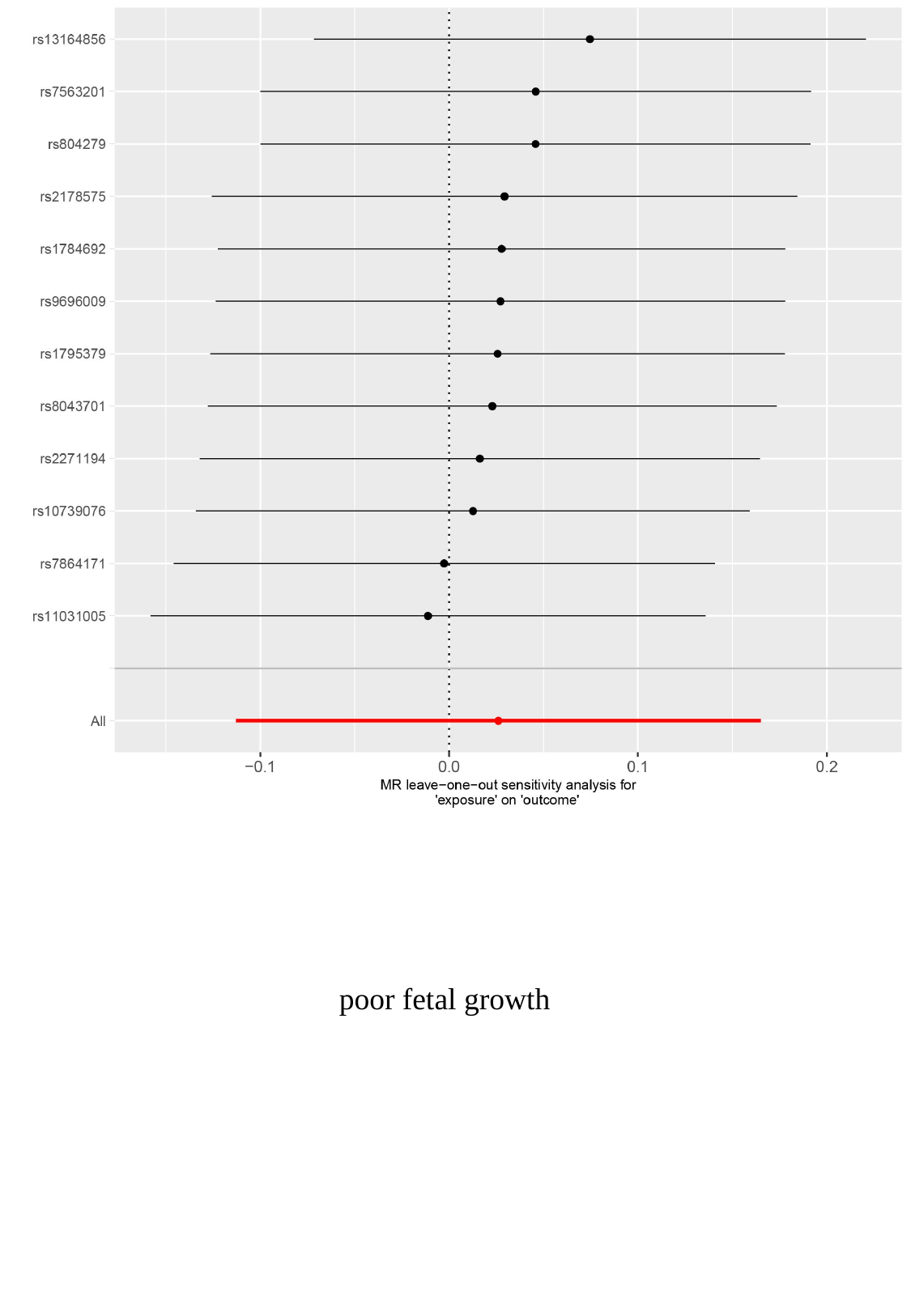

poor fetal growth
